# Supplementary material for: Mental health concerns and needs of international students in higher education settings: A scoping review protocol
Source: PLoS One. 2025 Aug 8;20(8):e0327812. doi: 10.1371/journal.pone.0327812 (PMC12334020; doi:10.1371/journal.pone.0327812)
Supplement: S2 File — (DOCX) [file pone.0327812.s002.docx]

The search strategy used for Ovid MEDLINE(R) ALL <1946 to March 26, 2024> is outlined below:

1 mental health/ or mental disorders/ or anxiety disorders/ or neurotic disorders/ or panic disorder/ or exp depressive disorder/ or Resilience, Psychological/ or exp Adaptation, Psychological/ or mental fatigue/ 534726

2 self mutilation/ or exp suicide/ or exp stress, psychological/ 232357

3 ((mental or mood or psychiatric* or psychologic* or emotion* or social* or eudaimonic or affective or psychopathologic* or social) adj2 (disorder* or health or hygiene or illness* or wellbeing or "well being" or "well−being" or welfare or "well-fare" or wellness or factor* or disease* or dysfunction* or disturbance* or state* or syndrome* or adjustment* or attitude* or stigma* or disparit* or exhaustion or resilience* or adaptation*)).ab,kf,ti. 633780

4 (depress* or suicid* or MDD or "self mutilation" or "self harm*" or dysphori* or dysthymi* or melancholi* or automutilat* or "self-harm*" or "self-injur*" or "self injur*").ab,kf,ti. 676859

5 (distress* or dystress* or stress* or GAD or panic or anxiet* or neurotic* or psychoneuroses or psychoneurosis or neuroses or neurosis or psychasthenia or burnout*).ab,kf,ti. 1546058

6 1 or 2 or 3 or 4 or 5 2609618

7 exp Students/ 174398

8 exp "emigrants and immigrants"/ or refugees/ or "transients and migrants"/ 41697

9 acculturation/ or cross-cultural comparison/ or cultural diversity/ or diversity, equity, inclusion/ or exp internationality/ or exp culture/ or "ethnic and racial minorities"/ or minority groups/ 365037

10 8 or 9 397154

11 7 and 10 9592

12 (student* adj4 (international* or global or foreign* or oversea* or immigrant* or "non native" or "non domestic" or emmigrant* or abroad or cultur* or refugee* or migrant* or expatriate*)).ab,kf,ti. 7529

13 (student* and (acculturat* or assimilat* or transcultural or globalization or multinational or intercultural or crosscultur*)).ab,kf,ti. 2341

14 11 or 12 or 13 16911

15 universities/ or academia/ or exp education, professional/ 383685

16 Foreign Medical Graduates/ 3639

17 (education adj3 (higher or postsecondary or tertiary or academia or continuing)).ab,kf,ti. 55168

18 (universit* or colleg*).ab,kf,ti. 626564

19 15 or 16 or 17 or 18 963258

20 6 and 14 and 19 2009
