# Supplementary material for: Mental health concerns and needs of international students in higher education settings: A scoping review protocol
Source: PLoS One. 2025 Aug 8;20(8):e0327812. doi: 10.1371/journal.pone.0327812 (PMC12334020; doi:10.1371/journal.pone.0327812)
Supplement: Attachment — Submitted filename: Scoping Review Protocol_PLOSOne_Aug 2024_Clean Version.docx [file pone.0327812.s003.docx]

STUDY PROTOCOL

**Mental health Concerns and Needs of International students in Higher education Settings: A Scoping Review Protocol**

**Kira Rudakova^1,2^ and Shalini Lal^1,2,3^***

**1** School of Rehabilitation, Université de Montréal, Montréal, Québec, Canada, **2** Youth Mental health and Technology Lab, Université de Montréal Hospital Research Centre, Montréal, Québec, Canada, **3** Douglas Research Centre, Montréal, Québec, Canada

* [shalini.lal@umontreal.ca](mailto:shalini.lal@umontreal.ca)

# Abstract

## Introduction

The transition to higher education is a significant milestone for many individuals. However, it also brings new stressors and challenges, particularly for international students adjusting to life in a foreign country. Despite the increasing diversity of student populations globally, there's a notable lack of comprehensive reviews on the mental health concerns and needs of international students. Existing reviews on the mental health and psychosocial adjustment of international students often concentrate on acculturation stress or disproportionately focus on specific regions or student populations. ~~However,~~ this narrow emphasis limits the comprehensive understanding of international students' mental health and well-being. **What factors from mental health? What are the links between your main theme or focus and other ‘not primary’ aspects? The transitions should be better detailed and worked;**

## Objective

The objective of this scoping review is to map and synthesize the research literature on the mental health concerns and needs of international students in higher education settings. **What are the applications for “map and synthesise”? What health concerns? What needs? Please detail and work on overall and other specific**

## Methods

A search strategy guided by the JBI **abbreviations** Manual of Evidence Synthesis has been developed according to the PCC **abbreviations** framework and will be applied to four electronic databases (i.e., MEDLINE, Embase, PsycInfo, and CINAHL). The selection strategy for abstract and title and full-text screenings will follow the same procedures **redundant**: initially, the strategy will be piloted by two reviewers on subsets of 10 articles until 90% agreement rate among reviewers is achieved. **It’s not clear. Please justify.** Once this agreement rate is reached, the remaining articles will be screened **independently by a single reviewer**. **I understood, but this raises the risk of bias too. Maybe it’s important to maintain the original procedure.** The data extraction will be piloted by two reviewers on a subset of 5 included studies, after which one reviewer will independently proceed with extraction process. Main findings will be presented through descriptive statistics, using tables and figures. **There is no mention of the eligibility criteria, the search workflow, how extraction and subsequent parts will be performed, what kind of parameters for stats, and what variables and outcomes will be presented**

## Expected Contributions

This scoping review will comprehensively assess existing literature on the mental health needs and experiences of international students, ~~thus~~ shedding light on overlooked issues and identifying areas for further research and interventions. **Please consider refining providing the potential implications and how the study will be important.**

# Introduction

The transition to higher education signifies a ~~pivotal~~ moment for many individuals, representing the beginning of academic endeavors, new experiences, and personal development [1,2]. Higher education in this context encompasses learning institutions such as universities and colleges that ~~award academic degrees or professional certifications, including both undergraduate and graduate~~ studies [3]. However, this transition to higher education also represents a new set of challenges and stressors that can have a significant impact on student mental health and well-being. From managing an academic ~~workload to navigating~~ new responsibilities [4,5], university students are burdened with increased levels of stress, anxiety, and depression [6], which subsequently impacts their academic performance and ability to complete their degree. As such, investigating student mental health and factors associated with it ~~have~~ **has** become a prominent focus in higher education research [7,8].

While the challenges of higher education are universal, international students face additional layers of complexities as they navigate life in a foreign country, which can exacerbate existing mental health concerns or give rise to new ones. For instance, the experience of being far from home and family support systems can trigger feelings of homesickness and adjustment difficulties, which may manifest as symptoms of depression or emotional distress [9-11]. Potential language barriers may further ~~compound~~ these feelings of isolation and difficulty in forming social connections, leading to worsened levels of loneliness and anxiety [12,13]. Finally, cultural differences in academic expectations and teaching styles may result in academic stress and performance anxiety among international students, especially if they feel pressure to excel in unfamiliar educational environments [12]. These are just a few examples of the ~~myriad~~ ways in which international students may experience mental health concerns while pursuing their higher education abroad.

With these complexities in mind, it is not surprising that recent years have seen a significant rise in mental health concerns among the international student population [14]. Studies have shown that international students often experience higher levels of stress, anxiety, and depression, even when compared to their domestic peers [15-17]. Despite the rising prevalence of these mental health challenges among international students, there remains a scarcity of literature reviews, including systematic reviews, that comprehensively address these issues. Existing literature often focuses on specific aspects of international student experiences, such as acculturation, without providing an in-depth analysis of their mental health status and needs. For instance, reviews by Smith and Khawaja [12] and Alharbi and Smith [18] primarily discuss predictors of psychosocial adjustment but only briefly mention mental health outcomes, doing so from an acculturation standpoint (i.e., acculturation stress). Although acculturation can play an important role in psychosocial adjustment, it should not be assumed as the default reason for all mental health-related challenges faced by international students. Furthermore, some reviews [19, 20] including a scoping review by McKenna et al. [21], which explores challenges faced by international students in health professions, tend to focus on the negative factors that impact student wellbeing. These reviews do not sufficiently address the positive factors that can enhance mental health, such as social support, resilience, and effective coping strategies, thus potentially overlooking ~~nuanced~~ aspects of international student mental health experiences. **The paragraphs shift abruptly, and the transitions are not smooth. Additionally, the text contains some contradictions. There is a need to provide a more in-depth interpretation of previous findings and the background, followed by a clear explanation of the importance of your study**

Based on a preliminary scan of the literature on international student mental health, it seems that there is a prominent trend of disproportional focus on specific populations. Such focus includes East~~-~~Asian students [22,10], specific regions, such as the United States [23], and specific disciplines, like medicine and nursing [24, 21, 20]. A systematic review by Zhang and Goodson [10] exemplifies this trend, providing a comprehensive analysis of predictors for the psychosocial adjustment of international students in the United States, yet predominantly focusing on East Asian students. Similarly, Li et al. [25] conducted a systematic review offering a multifaceted insight into the psychological well-being of international students, but again, their review primarily centers on East Asian students. Meanwhile, the systematic review by Brunsting et al. [26] on the predictors of undergraduate international student psychosocial adjustments exclusively focuses on universities in the United States. By focusing on specific cultural or regional groups, these reviews risk ~~neglecting~~ the diverse experiences among international students worldwide. International students come from a variety of cultural backgrounds, each with its own set of values, norms, and challenges related to mental health. As a result, overlooking this diversity limits our understanding of the cross-cultural experiences of international students.

Furthermore, a comprehensive understanding of international student mental health requires consideration of the host country's cultural context. Different host countries have distinct educational systems, social climates, and support services available to international students, all of which can significantly impact their mental well-being [22]. By exclusively focusing on specific regions like the United States, researchers may fail to capture the unique stressors and resources present in other host countries, thereby limiting the applicability of their findings on a global scale. Therefore, a more comprehensive review that encompasses a diverse range of cultural backgrounds and host countries is needed for a ~~holistic~~ understanding of international student mental health concerns, needs, and experiences. Through such research, common stressors and protective factors across cultures will be identified, and unique challenges faced by students in different contexts will be highlighted.

As demonstrated, there are currently few literature reviews that have comprehensively reported mental health concerns and needs of international students, particularly in relation to their experiences and challenges in higher education settings. This gap in the literature highlights the critical need for the present scoping review, the purpose of which is to systematically map the existing literature on mental health among international students. This will help to identify common themes and trends across studies, ~~shedding~~ light on the most prevalent issues faced by international students in higher education settings. Through this, future research and policy makers can ~~tailor~~ interventions and support services that are not only culturally sensitive but also effective in addressing the diverse needs of international students, ultimately enhancing international student well-being and academic success.

Numerous factors informed the decision to opt for scoping review methodology. First, a scoping review methodology allows for a broad exploration of the existing literature, encompassing various study designs, methodologies, and geographic locations. Given the diverse backgrounds and experiences of international students worldwide, this approach is useful for capturing the breadth and depth of research on their mental health. Second, by adopting a scoping review approach, we seek to identify gaps and limitations in the existing literature systematically, which is necessary for feasibility evaluation of more targeted studies that address specific aspects of international student mental well-being and eventually the development of evidence-based interventions tailored to the unique needs of international students. **Provide specific examples of cultural challenges or stressors faced by various groups of international students. Cite studies that overlook critical aspects of their experiences. Additionally, provide evidence to support this critique. Many sentences are lengthy and contain multiple ideas, which makes the text harder to follow. Breaking these sentences into focused statements will refine the text.**

Objective and Review Questions

**Why is this section presented as a separate topic? The objectives and hypotheses are discussed throughout the main text without clear headings or sections. This lack of organisation can lead to confusion for the reader.** The primary objective of this scoping review is to comprehensively map and report on the mental health concerns and needs of international students enrolled in higher education institutions globally. The secondary objective of this scoping review is to explore the cultural, social, and institutional factors that influence the mental health of international students pursuing higher education.

The research questions below have been informed by the primary objective of the review, as well as the population, concept, and context (PCC) framework proposed by the Joanna Briggs Institute (JBI) manual of Evidence Synthesis [27]:

1. What are the mental health concerns experienced by international students in higher education settings?
2. What are the cultural, social, and institutional factors influencing the mental health of international students pursuing higher education?

**The research questions are too broad and need more focus. Narrowing them will help the address issues in greater depth. Another option is to detail or extend a little bit. The objectives should also be clearer and more precise to give the study a clear path.**

# Eligibility Criteria

The population, concept, and context (PCC) framework proposed by the JBI Manual for Evidence Synthesis [27] will inform the inclusion and exclusion criteria of this scoping review.

Population. The population of focus will be international students enrolled in higher education institutions globally, including undergraduate and graduate levels, across various academic disciplines. International students, for the purpose of this scoping review, refer to individuals enrolled in higher education institutions outside of their home country, typically on a temporary visa or refugee status [28]. Synonyms such as “global”, “foreign”, “overseas”, “abroad”, and “cross-cultural” will be considered to ensure a comprehensive exploration of the diverse experiences of international students. Studies that include both international students and domestic students will be considered, as long as there is a clear distinction between the two populations (i.e., studies that draw a comparison between international students and domestic students).

Studies that focus on international student populations enrolled in short-term exchange studies abroad will be excluded, as there is not enough time to address the transition period and its effects on mental health in such programs [29]. Studies that do not differentiate samples of international students studying abroad versus those returning to their home countries in their analysis will also be excluded.

The review will not be restricted by nationality, citizenship, or specific demographic characteristics (i.e. gender, age, sexuality), allowing for a broad understanding of the mental health concerns and needs within this dynamic population.

Concept. Mental health encompasses a person's emotional, psychological, and social well-being, influencing how individuals think, feel, and behave [30]. Importantly, mental health is not merely the absence of mental disorders but also includes the ability to manage life's challenges, build healthy relationships, and achieve personal goals.

In this scoping review we will focus on the dimensions of “mental health” and “well-being", as well as mental health conditions and mental health disorders. Mental health conditions are a broad term that includes a range of emotional and psychological challenges that may affect well-being, including anxiety, depression, and stress. Meanwhile, mental health disorders are clinically diagnosed conditions that significantly impair cognitive, emotional, or social abilities, such as generalized anxiety disorder (GAD) and major depressive disorder (MDD) [31]. To ensure a comprehensive understanding of international student mental health, we will not exclude studies based on specific mental health disorders or their severity. Furthermore, studies that do not solely focus on mental health or well-being but include it as one of the clearly outlined outcomes will also be included. For instance, studies that explore both mental health and academic performance of international students will be considered eligible. Additionally, we will consider studies assessing occupation-based mental health aspects, such as performance anxiety, as these contribute to understanding the intricacies of mental health in various contexts.

Context. The context for this scoping review will center around higher education institutions globally. Higher education in this context includes various learning institutions that award academic degrees or professional certifications, such as universities, colleges, and various professional schools in disciplines such as law, medicine, and technology where international students can pursue full-time studies [3].

Considering the different use of terminology globally, such as "university" versus "college," it is important to examine the full text to understand what "college" signifies. For example, in the Quebec context, "college" could refer to the College of General and Professional Teaching (CEGEP), which is not a higher education context. Therefore, studies that mention "college" but do not pertain to a higher education context will be excluded upon full-text review. Additionally, non-traditional higher education settings such as language schools, vocational schools, adult education centers, and online learning platforms (e.g., Coursera, Udemy, Khan Academy) will be excluded, as they may have different academic structures and student demographics to the focus of this scoping review.

The review will not be restricted by geographical location of the higher education institutions to capture diverse cultural contexts and experiences. This approach will allow us to identify common challenges across different locations, which will enhance relevance and impact of our findings.

Any study that does not meet the eligibility criteria outlined above will be excluded from the scoping review.

Types of Sources of Evidence. Additional to the PCC framework, this scoping review has other eligibility criteria. First, only materials available in English and French will be included in the review as both reviewers are proficient in these languages. Second, no year limit is applied to capture the historical perspectives as well as the recent developments in international student mental health. Third, secondary sources (e.g., meta-analysis and systematic reviews) and grey literature (e.g., conference presentations) will be excluded from the review. In cases of secondary sources meeting the PCC inclusion criteria, the sources will be retained, and the studies reviewed in them will be screened. Any study that does not meet the eligibility criteria outlined above will be excluded from the scoping review.

**The section provides some exclusion criteria, such as excluding studies on short-term exchange students, but it needs to specify other types of studies that may not be relevant, like those focussed only on other aspects (e.g. physical health) without focusing on mental health. Since mental health is broadly defined, clarify whether a reference to it in a study is enough for inclusion. Please consider other aspects, like cultural.**

**The criteria feel scattered throughout the text, making harder to follow. Streamlining and organising this section more clearly will help readability. Also, some details (e.g. distinction between “college” and “university” terminology) seem unnecessary and don't contribute much to the main objectives.**

# Methods

The proposed scoping review (registered in OSF: [osf.io/kh9dc)](https://osf.io/kh9dc) will be conducted in accordance with the JBI Manual for Evidence Synthesis [27], recommendations of Levac et al. [32] and will be reported in accordance with PRISMA-ScR guidelines [33] and PRISMA-P checklist (S1 File) [34] (<https://www.prisma-statement.org/scoping>). **Expand on how these will be integrated or operationalised. This part is important but lacks detail on how all the guidelines and recommendations will work together.**

## Sources of Evidence

Four electronic databases (i.e., MEDLINE through Ovid, Embase through Ovid, PsycInfo through Ovid, and CINAHL through EBSCO) **Why CINAHL? Seems outside the topic.** will be examined using distinct search strategies, to ensure thoroughness and comprehensiveness in addressing the proposed research topic. MEDLINE and Embase were selected because of their extensive coverage of biomedical literature, providing access to clinical studies relevant to mental health concerns experienced by international students in higher education contexts. PsycInfo was selected for its focus on psychology and behavioral sciences literature, offering insights into the cultural and social factors that influence international student mental health. Finally, CINAHL was selected for its focus on nursing and allied health literature, which includes research on the mental health of healthcare university students and various factors, including institutional factors, that influence their mental well-being, thus providing valuable insights applicable to understanding similar factors affecting international students. The reference list of primary and secondary sources, identified as relevant to the topic of inquiry, will be examined. **The selection of databases could be more targeted to the research question by including education-focused and region-specific sources.**

## Search Strategy

The first author (KR) collaboratively developed the search strategy with guidance and input from the senior author (SL) and the Université de Montréal librarian (SC). The senior author and the first author incorporated the scoping review objectives and the population, concept, and context (PCC) framework [27] into the initial search strategy. The librarian then provided input on refining the strategy, leading to further refinements based on a detailed examination of preliminary search results.

As the first step of the search strategy development, a preliminary search on the database MEDLINE was conducted. Following this, index terms used to categorize publications (i.e., descriptors), along with key words pertaining to the publication abstract, title and headings were identified and selected. The selected index terms and key words informed the development of a complete search strategy for MEDLINE (S2 File). This search strategy will be adapted for the three other electronic databases (i.e., Embase, PsycInfo, and CINAHL). Along with the search strategy, the reference list of relevant secondary sources will be screened to identify potential articles that should be included in this scoping review. **Details on how terms will be adjusted for different databases are important. Also detail sensitivity and specificity. What strategies, boolean, etc? The search strategy appears static, with no indication that the authors will adjust the search strategy based on evolving literature or emerging themes during the screening. Consider using a more flexible approach or strategy.**

## Study Selection

The study selection of articles appropriate for the proposed scoping review will be conducted by two reviewers and will be guided by the inclusion and exclusion criteria outlined above. The citations obtained from the databases search will be initially imported into EndNote [35] for manual duplicate and grey literature removal by the first author. Following this, the citations will be imported into Covidence, a web-based collaborative software platform commonly used to assist in knowledge synthesis [36], where any remaining duplicates will be automatically removed.

As proposed in the JBI Manual of Evidence Synthesis [27], the study selection will first be piloted on a random subset of studies to ensure that both reviewers have a clear understanding of the eligibility criteria and selection process. Subsets of 10 citations will be independently screened by two reviewers. Any discrepancies in the inclusion of studies will be resolved through discussion and consensus between the reviewers, and if needed, a third reviewer will be consulted. Pilot testing will be deemed successful, and the study selection process will commence once a minimum 90% agreement rate between reviewers is achieved.

The study selection process will proceed with two main stages: first, title and abstract screening, followed by full-text review. During title and abstract screening, studies will be assessed based on the eligibility criteria to determine their relevance to the research question. Subsequently, full-text review will involve a more detailed examination of the selected studies to confirm their eligibility for inclusion in the scoping review. Abstracts that do not contain sufficient information to assess eligibility will undergo full-text review. Only the first author will be involved in the screening process at each stage. In cases where there is uncertainty about the eligibility of an article, the first author will consult with the second reviewer. If a consensus cannot be reached, a third reviewer will be consulted to make the final decision, ensuring thoroughness and impartiality in the selection process. The third reviewer will conduct full-text review to determine the article's eligibility for inclusion in the scoping review, ensuring thoroughness and impartiality in the selection process.

**The use of a small sample for piloting the eligibility criteria may not be representative of the variability in the studies retrieved. Please provide more detailed guidelines on how disagreements will be managed (e.g. for involving the third assessment, documentation, etc.) Expanding the pilot test to include a larger sample (e.g. 20–30 citations) would provide more robust insights into potential challenges in applying the eligibility criteria.**

## Data Extraction

Following the conclusion of the study selection, data extraction process will begin. First, a charting tool will be developed to facilitate the extraction process and ensure its validity. The charting tool will outline categories of information to be extracted from the included studies and will be organized in Covidence.

Initially, the legend categories will be informed by the data categories listed in the JBI Manual of Evidence Synthesis [27], such as: title, Digital Object Identifier (DOI), author(s), year of publication, country of origin, sample size, study design, objective(s), and key findings. Furthermore, the categories within the charting tool will be informed by the research questions and the eligibility criteria of the scoping review. Considering the population component of the PCC framework, data related to the study sample will be extracted, such as sociodemographic characteristics (i.e., age, gender, country of origin, socioeconomic status) and levels and focus of studies (e.g., undergraduate, graduate studies). **The proposed charting tool may miss themes important for understanding the main outcomes. Scoping reviews aim to cover a broad literature, including unexpected findings. However, the authors didn’t explain how handle studies outside predefined categories.**

For the concept component of PCC, data extraction will encompass various dimensions of international student mental health. This includes identifying mental health status and concerns (e.g., depression), along with the methods used to assess these concerns, distinguishing between quantitative measurement tools (e.g., Beck Depression Inventory) and qualitative approaches (e.g., interviews, open-ended survey questions). Furthermore, negative factors affecting mental health (e.g., social isolation) and positive factors contributing to mental well-being (e.g., institutional support) will also be identified during data extraction.

Finally, for the context component of PCC, information pertaining to higher education setting, such as types of educational institution (e.g., university, college) and the country or regions where the educational institution is located will be extracted. **The strategy for categorising contextual factors is also too simplified. Higher education varies greatly between countries, and broad categories like "university vs. college" or general won’t gather these complexities. This could limit to reflect the diverse realities of these institutions. Separating data on mental health concerns and assessment methods raises concerns about comparability. Quantitative data can be standardised, but qualitative data requires careful interpretation. The method for integrating both types of data is unclear, which may result in inconsistent analysis of qualitative findings.**

The data extraction tool will remain adaptable, subject to iterative refinement in response to emergent findings. As recommended by the JBI Manual of Evidence Synthesis [27] and Levac et al. [32], the data charting table will be piloted on a random sample of included studies to ensure that the tool is comprehensive and easy to use. Two independent reviewers will be involved in the charting table pilot of five included studies. Once the pilot is successful, the first author will proceed with data extraction independently. If needed, the author will consult the other reviewer to resolve any uncertainties or discrepancies during the extraction process. **The tool collects basic details like sample size and study design, but won’t address how the quality of the studies will be considered. It’s common to not consider this in scoping reviews, but mentioning differences in study designs is important.**

## Data Synthesis and Presentation

The extracted data will be summarized and presented using descriptive statistics (e.g., frequencies and percentages). Tables will be used to visually represent a comprehensive overview of various mental health concerns reported by international students. The first table will include data on types of mental health concerns reported by international students (e.g., depression) and the prevalence of each mental health concern among international students. A second table will incorporate sociodemographic information extracted from the studies and whether there is an association between certain sociodemographic factors and mental health concerns among international students. This table may include details such as age, gender, country of origin, level of education, field of study, and any observed correlations with specific mental health issues. Finally, a third table will include negative and positive factors reported by international students that affect their mental health and well-being (e.g., lack of social support).

Additional findings may be reported based on identification of patterns among the results obtained and their relevance to the objectives of this scoping review. For instance, if a distinct pattern emerges related to mental health concern differences between domestic and international student or utilization of university support resources by international students, such findings will warrant their own dedicated tables for comprehensive analysis.

Moreover, additional forms of visual presentation might be needed to convey complex patterns or relationships identified in this scoping review, such as charts and graphs. These visual aids will enhance the understanding of the data.

# Limitations

To our knowledge, this scoping review will be one of the first to synthesize and comprehensively report on mental health of international students in higher education. However, this scoping review is not without its limitations.

First, it is important to highlight that extensive efforts were made to include comprehensive key terms in the search strategy, related to mental health and well-being, mental illness, as well as more specific mental health conditions, such as stress, anxiety, and depression. However, other terms and concepts related to mental health conditions (e.g., schizophrenia, bipolar disorder) were not included in the search strategy, as the intention is to focus on the most common and prevalent mental health concerns among international students, aiming to balance comprehensiveness with feasibility. As a result, there is a possibility that studies focusing on mental health conditions beyond those explicitly mentioned in the search strategy may be inadvertently excluded in this scoping review.

Second, this scoping review will not assess methodological rigour of the included studies. While such evaluation could provide valuable insights into the reliability and validity of collected evidence, the primary objective of this scoping review is to map the existing literature and identify key themes when it comes to mental health of international students. As such, the impact of this scoping review may be limited in informing the selection of evidence-based interventions or in making specific recommendations for practice or policy in the field of international student mental health.

Third, grey literature and secondary sources, including unpublished studies and conference abstracts, will not be considered in this scoping review. This decision was made due to concerns about the validity of grey literature, as these sources are not peer-reviewed and may include preliminary results that could differ from the final published results. Consequently, there is a possibility that relevant studies might go undetected and not be included in the scoping review, thus leading to an incomplete representation of literature landscape.

Finally, the scope of this review is limited to studies published in English and French. While this choice aims to be as inclusive as possible within the constraints of the reviewers' language proficiencies, it inevitably excludes research published in other languages. This language limitation may contribute to an overemphasis on certain geographic regions and student populations, reiterating the existing research biases and potentially overlooking relevant studies from non-English and non-French speaking contexts.

# Conclusion

Despite the outlined limitations, this scoping review aims to comprehensively map the available evidence on mental health among international students and highlight the gaps in current research, particularly the overemphasis on certain geographic regions and student populations. Through the synthesis of existing literature and key themes, this review will provide valuable insights into types of mental health concerns experienced by international students. Additionally, it will identify the factors that both negatively and positively impact their mental well-being, offering a more nuanced understanding of the challenges and supports related to their mental health.

This scoping review will pave the way for future research to address these gaps and build upon the existing knowledge base, thus ensuring more inclusive and representative studies. This review seeks to contribute significantly to the field by uncovering areas for future research and informing policy and practice to better support the mental health needs of international students Furthermore, this synthesis of knowledge can provide a base and guidance for conducting a systematic review and meta-analysis on the topic of inquiry. Finally, the results of this scoping review may inform the development of targeted interventions and support services tailored to the diverse needs of the international student population.

**The plan for data synthesis and presentation is solid but could be refined with a detailed approach to integrating qualitative data, exploring correlations, and highlighting complex patterns. The limitations section would be stronger by addressing challenges like study quality, publication bias, and heterogeneity in the included studies.**

# Supporting Information

**S1 File. PRISMA-P Checklist.**

(DOCX)

**S2 File. Detailed search strategy for MEDLINE.**

(DOCX)

# Acknowledgments

The authors would like to acknowledge and thank Sarah Cherrier, a librarian at the Université de Montréal, for her invaluable assistance and support in refining the search strategy outlined in this protocol. Additionally, we extend our gratitude to Ayda Khalili, a research intern at the Youth Mental health and Technology (YMHTech) Laboratory, for her input in validating the citations included in the protocol and Tania Sabatino, a Planning, Programming and Research Officer at the YMHTech Laboratory, for reviewing the protocol.

# Author Contributions

**Conceptualization:** Kira Rudakova, Shalini Lal.

**Investigation:** Kira Rudakova.

**Supervision:** Shalini Lal.

**Writing – original draft:** Kira Rudakova.

**Writing – review and editing:** Shalini Lal.

**Project Administration:** Kira Rudakova, Shalini Lal.

**Resources**: Shalini Lal.

# References

Ensure adherence to Vancouver and that all refs are consistent in formatting and punctuation

1. Cohen P, Kasen S, Chen H, Hartmark C, Gordon K. Variations in patterns of developmental transmissions in the emerging adulthood period. Developmental Psychology. 2003;39(4):657-69. doi:10.1037/0012-1649.39.4.657. PMID: 12859127.
2. Schulenberg JE, Sameroff AJ, Cicchetti D. The transition to adulthood as a critical juncture in the course of psychopathology and mental health. Development and Psychopathology. 2004 Dec;16(4):799-806. doi:10.1017/S0954579404040015. PMID: 15704820.
3. Britannica, T. Editors of Encyclopaedia. Higher education. Encyclopedia Britannica [Internet] 2024. Available from: <https://www.britannica.com/topic/higher-education>
4. Sheldon E, Simmonds-Buckley M, Bone C, Mascarenhas T, Chan N, Wincott M, et al. Prevalence and risk factors for mental health problems in university undergraduate students: A systematic review with meta-analysis. Journal of Affective Disorders. 2021 May ;287(1):282-92. doi:10.1016/j.jad.2021.03.054. PMID: 33812980.
5. Brown JSL. Student mental health: some answers and more questions. Journal of Mental Health. 2018 May 4;27(3):193-6. doi:10.1080/09638237.2018.1470319.
6. Storrie K, Ahern K, Tuckett A. A systematic review: Students with mental health problems—A growing problem. International Journal of Nursing Practice. 2010 Feb;16(1):1-6. doi:10.1111/j.1440-172X.2009.01813.x. PMID: 20158540.
7. Abrams Z. Student mental health is in crisis. Campuses are Rethinking Their Approach. Monitor Psychol [Internet]. 2022;53(7). Available from: <https://www.apa.org/monitor/2022/10/mental-health-campus-care>.
8. Campbell F, Blank L, Cantrell A, Baxter S, Blackmore C, Dixon J, et al. Factors that influence mental health of university and college students in the UK: a systematic review. BMC Public Health. 2022;22(1):1778. doi:10.1186/s12889-022-13943-x. PMID: 36288256.
9. Prieto-Welch, S.L. (2016), International Student Mental Health. Student Services, 2016 Dec; 53-63 doi:10.1002/ss.20191.
10. Zhang J, Goodson P. Predictors of international students’ psychosocial adjustment to life in the United States: A systematic review. International Journal of Intercultural Relations. 2011 Mar;35(2):139-62. doi:10.1016/j.ijintrel.2010.11.011.
11. Wang KT, Wei M, Zhao R, Chuang C-C, Li F. The Cross-Cultural Loss Scale: Development and psychometric evaluation. Psychological Assessment. 2015 Mar;27(1):42-53. doi:10.1037/pas0000027. PMID: 25384069.
12. Smith RA, Khawaja NG. A review of the acculturation experiences of international students. International Journal of Intercultural Relations. 2011 Nov;35(6):699-713. doi:10.1016/j.ijintrel.2011.08.004.
13. Mesidor JK, Sly KF. Factors that Contribute to the Adjustment of International Students. Journal of International Students. 2016 Jan;6(1):262-82. doi:10.32674/jis.v6i1.569.
14. Forbes-Mewett H, Sawyer A-M. International Students and Mental Health. Journal of International Students. 2016 Jul;6(3):661-77. doi:10.32674/jis.v6i3.348.
15. Janca A, Helzer JE. Psychiatric Morbidity of Foreign Students in Yugoslavia: a 25 Year Retrospective Analysis. International Journal of Social Psychiatry. 1992 Dec;38(4):287-92. doi:10.1177/002076409203800407. PMID: 1477841.
16. Henning MA, Hawken SJ, Krägeloh C, Zhao Y, Doherty I. Asian medical students: quality of life and motivation to learn. Asia Pacific Education Review. 2011 Jan;12(3):437-45. doi:10.1080/10401334.2012.715261.
17. King N, Rivera D, Cunningham S, Pickett W, Harkness K, McNevin SH, et al. Mental health and academic outcomes over the first year at university in international compared to domestic Canadian students. Journal of American College Health. 2023;71(9):2663-7. doi:10.1080/07448481.2022.2078615. PMID: 35603953.
18. Alharbi E, Smith A. Review of the Literature on Stress and Wellbeing of International Students in English-Speaking Countries. International Education Studies. 2018 May;11:22. doi:10.5539/ies.v11n6p22.
19. Mori SC. Addressing the Mental Health Concerns of International Students. Journal of Counseling & Development. 2000 Apr;78(2):137-44. doi:10.1002/j.1556-6676.2000.tb02571.x.
20. Malecha A, Tart K, Junious DL. Foreign-Born Nursing Students in the United States: A Literature Review. Journal of Professional Nursing. 2012 Jul ;28(5):297-305.

doi:10.1016/j.profnurs.2012.03.002.

21. McKenna L, Robinson E, Penman J, Hills D. Factors impacting on psychological wellbeing of international students in the health professions: A scoping review. International Journal of Nursing Studies. 2017 Sep;74:85-94.

doi:10.1016/j.ijnurstu.2017.06.007. PMID: 28662365.

22. Candel O. Acculturative stress among international students-A literature review. Teachers and students in multicultural environments. 2021:199-220. doi:10.37384/001c.23392.
23. Cao Q-T, Vuong Q-H, Pham H-H, Luong D-H, Ho M-T, Hoang A-D, et al. A Bibliometric Review of Research on International Students’ Mental Health: Science Mapping of the Literature from 1957 to 2020. European Journal of Investigation in Health, Psychology and Education. 2021 Jul;11(3):781-94. doi:10.3390/ejihpe11030056.
24. Merry L, Vissandjée B, Verville-Provencher K. Challenges, coping responses and supportive interventions for international and migrant students in academic nursing programs in major host countries: a scoping review with a gender lens. BMC Nursing. 2021 Sep;20(1):174. doi:10.1186/s12912-021-00678-0. PMID: 34759065.
25. Li J, Wang Y, Xiao F. East Asian International Students and Psychological Well-Being: A Systematic Review. Journal of International Students. 2014 Oct;4(4):301-13. doi:10.32674/jis.v4i4.450.
26. Brunsting NC, Zachry C, Takeuchi R. Predictors of undergraduate international student psychosocial adjustment to US universities: A systematic review from 2009-2018. International Journal of Intercultural Relations. 2018 Sep;66:22-33.

doi:10.1016/j.ijintrel.2018.06.002.

27. Aromataris E, Munn Z. JBI Manual for Evidence Synthesis. JBI; 2020. Available from: [https://synthesismanual.jbi.global](https://synthesismanual.jbi.global/). doi:10.46658/JBIMES-20-01.
28. Statistics Canada. A changing portrait of international students in Canadian universities. Definition of International students 2011. Available from: <https://www150.statcan.gc.ca/n1/pub/81-004-x/2010006/def/intlstudent-etudiantetranger-eng.htm>
29. Titzmann PF, Lee RM. New temporal concepts of acculturation in immigrant youth. Child Development Perspectives. 2022 Sep;16(3):165-72. doi:10.1111/cdep.12458.
30. World Health Organization. Comprehensive mental health action plan 2013–2030. World Health Organization; 2013. Licence: CC BY-NC-SA 3.0 IGO.
31. World Health Organization. Mental disorders [Internet]. 2022. Available from: <https://www.who.int/news-room/fact-sheets/detail/mental-disorders>
32. Levac D, Colquhoun H, O'Brien KK. Scoping studies: advancing the methodology. Implementation Science. 2010;5(1):69. doi:10.1186/1748-5908-5-69. PMID: 20854677.
33. Tricco AC, Lillie E, Zarin W, O'Brien KK, Colquhoun H, Levac D, et al. PRISMA Extension for Scoping Reviews (PRISMA-ScR): Checklist and Explanation. Annals of Internal Medicine. 2018 Sep;169(7):467-73. doi:10.7326/M18-0850.
34. Moher D, Shamseer L, Clarke M, Ghersi D, Liberati A, Petticrew M, Shekelle P, Stewart LA. Preferred Reporting Items for Systematic Review and Meta-Analysis Protocols (PRISMA-P) 2015 statement. Syst Rev. 2015 Jan;4(1):1. doi:10.1186/2046-4053-4-1.
35. Bramer WM, Giustini D, de Jonge GB, Holland L, Bekhuis T. De-duplication of database search results for systematic reviews in EndNote. J Med Libr Assoc. 2016 Jul;104(3):240-3. doi:10.3163/1536-5050.104.3.014. PMID: 27366130.
36. Kellermeyer L, Harnke B, Knight S. Covidence and rayyan. J med libr assoc. 2018 Oct; 106(4):580. doi:10.5195/jmla.2018.513.
