# Supplementary material for: Mental health concerns and needs of international students in higher education settings: A scoping review protocol
Source: PLoS One. 2025 Aug 8;20(8):e0327812. doi: 10.1371/journal.pone.0327812 (PMC12334020; doi:10.1371/journal.pone.0327812)
Supplement: Attachment — Submitted filename: Response to Reviewers_Jan 16 2025.docx [file pone.0327812.s005.docx]

Dear PLOS ONE Editorial Team and Reviewers,

Thank you for your valuable feedback on our protocol. We appreciate the time and effort you have invested in reviewing our submission. Below, we address the comments received via email as well as those noted directly within the manuscript. To ensure clarity and organization in our response, we will first address the comments received via email, followed by the comments that were made directly in the manuscript, section by section.

Please note that references to changes made to the manuscript throughout this table correspond to the **Revised Manuscript with Track Changes**, which has been included as part of our resubmission.

| **Location of Comment** | **Reviewer Comment** | **Response** | **Revisions Made** |
| --- | --- | --- | --- |
| Email | Reviewer #1 expressed concerns about data availability, highlighting the PLOS Data policy, which states that all data underlying findings must be fully available without restriction at the time of publication. This includes providing data as part of the manuscript or depositing it in a public repository, with specific details about any restrictions | Thank you for your comment regarding the PLOS Data policy. We would like to clarify that this submission is a protocol for a scoping review, rather than a manuscript presenting review findings. As such, no data has yet been collected or analyzed, and there is currently no data available to share. The protocol outlines the planned methods for conducting the review. We are committed to adhering to PLOS’s Data policy, and once the scoping review is complete, any relevant data identified or used during the review will be made available in accordance with the policy.  To clarify this latter point, we have added a statement in the manuscript, section “Data Synthesis”, page 20. | Data Synthesis and Presentation:  The data underlying the findings will be made fully available without restriction within the final report or as supplementary files. |
| Email | Reviewer #1: Firstly, the authors themselves note that their review will not include terms related to more severe mental disorders, such as schizophrenia or bipolar disorder. While their rationale for focusing on common mental health issues is reasonable, excluding other disorders may lead to an incomplete picture and skewed results.  A second significant limitation is the need for more methodological assessment of the included studies. The review will only map the literature in evaluating the quality of individual studies, which significantly limits the ability to draw firm conclusions regarding the effectiveness of interventions or recommendations for health policy. Relying solely on collected data without assessing quality may lead to less valuable findings for practitioners.  Additionally, excluding grey literature, such as conference abstracts or unpublished studies, could be problematic, especially in mental health, where some innovative research may not yet have been published in traditional journals. This could lead to publication bias and the omission of valuable data.  One of the most significant limitations of the reviewed work is the restriction to studies published in English and French. Mental health concerns of international students are a global issue, and excluding literature in other languages (e.g., Chinese, Spanish, Arabic) could lead to the omission of essential studies from regions where the number of international students is significant. Such a language choice may result in an overemphasis on Western models of mental health, limiting the ability to draw conclusions that are universal for different cultural contexts. | We thank Reviewer #1 for highlighting the limitations we have written in the manuscript.   1. We acknowledge that our decision for our search strategy to focus on common concerns and disorders such as anxiety, depression, and stress may limit the scope of less common conditions represented in this scoping review. We have addressed this as a limitation in our protocol (see section “Limitations”, page 20). 2. In the Limitations section of our manuscript (see page 22), we have acknowledged that the lack of study quality assessment is a limitation. We will extract data on the study design (see section “Data Extraction”, page 16) and present this information in our findings, which should provide the reader with the context about methodologies used in the included studies. 3. As mentioned on page 21 of our protocol, the decision to exclude grey literature was made due to concerns about the validity of non-peer-reviewed sources, which also may present preliminary findings that differ from eventual published results. While we recognize that this may lead to publication bias and omit innovative research, we prioritized the inclusion of peer-reviewed studies to enhance the validity of our findings. 4. As stated in the Limitations section of our protocol (page 21), we recognize that restriction of reviewed studies only to English and French may lead to the omission of important research in other languages and could result in an overemphasis on Western models of mental health. Concurrently, it is important to note that our initial piloting of the search strategy managed to retrieve studies conducted in non-western contexts, such as Turkey, Jordan, Saudi Arabia, and Hong Kong, published in English. If these meet inclusion criteria, the final report will include studies from non-Western contexts. Nonetheless, the decision to exclude papers written in other languages was made to ensure feasibility and manageability of the review and we appreciate the need for a broader perspective on international student mental health across diverse cultural contexts. | N/A |
| Email | Reviewer #2: Are you going to achieve an agreement rate with the data extraction when moving from two reviewers to one? | We consulted the JBI Manual of Evidence Synthesis and Levac et al. for guidelines on the extraction process and concluded that we will not set an agreement rate for the extraction pilot. JBI Section 10.2.7 emphasizes the iterative nature of data extraction and recommends pilot testing the extraction form on a small number of studies, involving at least two reviewers. However, it does not prescribe setting a numerical agreement rate during the pilot phase; instead, JBI stresses the importance of ensuring transparency and refining the extraction process through collaborative review and discussion.  Similarly, Levac et al. advocate for two reviewers to independently extract data from an initial sample of studies and then meet to reconcile differences, with no mention of a numerical agreement rate between the reviewers.  Aligned with these guidelines, our extraction process will be as following:  Both reviewers will independently extract the data and compare each other's results. They will then meet to discuss any discrepancies to reach a consensus. Once the pilot is successful, the first author will proceed with data extraction independently.  We clarified this decision in the manuscript (section “Data Extraction”, page 18). | Data Extraction:  Both reviewers will independently extract the data and compare each other's results. They will then meet to discuss any discrepancies to reach a consensus. |
| Email | Reviewer #2: Expected contributions section, it isn’t clear from the introductory remarks that the current literature provides sufficient data to address ‘overlooked issues’? It would also help to specify, or provide examples, of what such overlooked issues might be. | We clarified in the introduction of our abstract the ‘overlooked issues’ that our scoping review aims to address.  We also reiterated those issues and implications of addressing those issues in the expected contribution section of the abstract (see page 3). | Introduction:  Existing reviews on the mental health and psychosocial adjustment of international students often concentrate on acculturation stress, thus overlooking other mental health concerns such as depression and anxiety, as well as positive mental health experiences like increased well-being. Meanwhile, other reviews tend to focus more heavily on specific regions, such as the United States and Australia, or student populations, particularly East-Asian students. While valuable, this focus may limit our understanding of the diverse mental health experiences of international students.  Expected Contributions:  This scoping review will assess existing literature on the mental health needs and experiences of international students, thus shedding light on overlooked issues, such as challenges beyond acculturation stress and the experiences of underrepresented student populations, including those studying outside of western countries. |
| Email | Reviewer #2: The argument that “Therefore, a more comprehensive review that encompasses a diverse range of cultural backgrounds and host countries is needed for a holistic understanding of international student mental health concerns, needs, and experiences.” is sound, but can you provide any preliminary data or evidence to suggest that studies already exist to review that would support this endeavour? | Our preliminary searches of the literature indicate that there are studies examining the experiences of international students from a variety of backgrounds and contexts. Examples include Safak-Ayvazoglu et al.'s investigation of Syrian refugees studying in Turkey, as well as research on international students in non-Western contexts such as Saudi Arabia (Alasmari, 2023), and Russia (Merenkov & Antonova, 2015). We added explicit mention of these studies and highlighted the importance of exploring research focused on diverse student populations and host country contexts in the manuscript (section “Introduction”, page 6). | Introduction:  While this research has provided valuable insights into these populations, it also underscores the opportunity to expand the scope of studies to more comprehensively reflect the diverse experiences of international students from a variety of cultural, regional, and institutional contexts [27-30]. For example, a qualitative investigation on assimilation of Syrian refugees studying in Turkey by Safak-Ayvazoglu et al. revealed that despite benefiting from Arabic language and the safety of their host country, these students still face significant mental health challenges due to trauma from war [27]. While certain aspects of their environment may facilitate adaptation, the enduring impact of their backgrounds and experiences can lead to complex mental health concerns. |
| Email | Reviewer #2: How will you consider, if at all, longitudinal studies that examine this topic comparing pre vs post enrolment. | Longitudinal studies that meet the Population, Concern, and Context (PCC) and Types of Sources of Evidence criteria outlined in the Eligibility Criteria section of the protocol will be included in this scoping review. Data from these studies will be extracted at each time point reported (e.g., at the start, mid-way, and completion) to ensure that changes over time are captured. For quantitative studies, numerical data such as prevalence rates, means, or percentages will be collected at each time point. For qualitative studies, evolving factors affecting mental health will be identified, and observations or themes reported by the authors will be extracted separately for each respective time point.    We added clarification regarding this inclusion in section “Data Extraction”, page 17 of the manuscript. | Data Extraction:  In the case of longitudinal studies, data will be extracted at each time point reported in the study (e.g., at the start, mid-way, and completion) to ensure that changes over time are captured. For quantitative studies, numerical data such as prevalence rates, means, or percentages will be collected at each time point. For qualitative studies, evolving factors affecting mental health will be identified, and observations or themes reported by the authors will be extracted separately for each respective time point. |
| Email | Reviewer #2: Will your analysis have the capacity to examine interactions between individual factors influencing student mental health? | As this is a scoping review, it will not conduct statistical calculations or analyses to examine interactions between individual factors, since scoping reviews are not intended for such analyses (JBI Manual of Evidence Synthesis, 2020, Section 10.2.8). However, if patterns or associations emerge during data extraction—such as a higher prevalence of anxiety in women or more reports of depression in students from certain countries—these will be reported as part of our findings. The analysis will focus on summarizing individual factors influencing student mental health, and any observed patterns across the extracted data will be presented descriptively. We added this specification in the Data Synthesis and Presentation section of our protocol (see page 19). | Data Synthesis and Presentation:  This scoping review will not conduct statistical calculations or analyses to examine interactions between individual factors, as scoping reviews are not typically intended for such analyses (JBI Manual of Evidence Synthesis, 2020, Section 10.2.8) [32]. However, during the data extraction process, any patterns or associations that emerge—for example, if anxiety is more frequently reported by women or if students from a particular country show higher rates of depression—will be identified and reported. |
| Protocol – Abstract – Introduction | What factors from mental health? What are the links between your main theme or focus and other ‘not primary’ aspects? The transitions should be better detailed and worked | We have addressed the comment by providing examples of mental health concerns beyond acculturative stress, as well as mentioning positive mental health experiences like increased well-being (see section “Abstract”, page 2). We also specified the overlooked issues related to geographic and student population focus. Given that this is an abstract, we are limited in terms of providing additional details. | The transition to higher education is a significant milestone for many individuals. However, it also brings new stressors and challenges, particularly for international students adjusting to life in a foreign country. Despite the increasing diversity of student populations globally, there remains a gap in existing reviews to capture fully international student mental health concerns and needs. Existing reviews on the mental health and psychosocial adjustment of international students often concentrate on acculturation stress, thus overlooking other mental health concerns such as depression and anxiety, as well as positive mental health experiences like increased well-being. Meanwhile, other reviews tend to focus more heavily on specific regions, such as the United States and Australia, or student populations, particularly East-Asian students. While valuable, this focus may limit our understanding of the diverse mental health experiences of international students. |
| Protocol – Abstract - Objective | What are the applications for “map and synthesise”? What health concerns? What needs? Please detail and work on overall and other specific | We revised the objective accordingly to specify the focus of this scoping review (see section “Abstract”, page 2): now it highlights the aim to expand our understanding of international students' experiences and needs by mapping summarize research on mental health experiences and concerns (e.g., depression) and factors influencing well-being (e.g., social support, institutional resources). | The objective of this scoping review is to map and summarize research on international students’ mental health experiences and concerns (e.g., depression) as well as factors influencing their well-being (e.g., social support and institutional resources). |
| Protocol – Abstract - Methods | Redundant. | Thank you for this comment. We have revised this section for conciseness. | Methods:  Two reviewers will pilot the selection strategy on subsets of 10 articles until a 90% agreement rate is achieved. Once this rate is reached, a single reviewer will screen the remaining articles independently. Two reviewers will then pilot the data extraction procedures on subsets of 10 included studies, after which one reviewer will proceed to extract the data independently. |
| Protocol – Abstract - Methods | I understood, but this raises the risk of bias too. Maybe it’s important to maintain the original procedure. | We acknowledge reviewer’s concern regarding the risk of bias. Our decision is based on feasibility of resources; however, we have implemented several strategies to mitigate the risk of bias and to ensure clarity of the inclusion/exclusion criteria to enhance reliability (see section Methods, page 15). We have also addressed this as a limitation in our protocol (see section “Limitations”, pages 21 and 22). | Limitations:  Fifth, there is a risk of bias in the selection process given that only one reviewer will be involved in most of this process (due to availability of resources). Nonetheless, several strategies are in place to reduce this risk including piloting multiple subsets of 10 articles until a 90% agreement is reached, and continuing to pilot until 3 additional consecutive sets also results in a minimum of 90% agreement. Moreover, a randomization generator for selecting citations will be employed to increase the likelihood of obtaining a diverse representation of studies in the pilot. |
| Protocol – Abstract - Methods | There is no mention of the eligibility criteria, the search workflow, how extraction and subsequent parts will be performed, what kind of parameters for stats, and what variables and outcomes will be presented. | We appreciate the reviewer’s concerns. These elements are discussed in the full protocol. However, we believe including these in the abstract would make it too lengthy, as the abstract is intended to provide a concise overview of the methodology. | N/A |
| Protocol – Abstract - Expected Contributions | Please consider refining providing the potential implications and how the study will be important. | We expanded on the potential implications of our scoping review, specifying which overlooked issues it may address, as well as how the findings may inform development of inclusive interventions and support resources (see page 3). | This scoping review will comprehensively assess existing literature on the mental health needs and experiences of international students, shedding light on overlooked issues, such as challenges beyond acculturation stress and the experiences of underrepresented student populations, including those studying outside of Western countries. Ultimately, the findings may identify areas for further research and inform educational institutions and mental health professionals in developing support resources that can effectively address diverse needs of international students. |
| Protocol – Revisions Throughout Introduction Section | One of the reviewers or editors included some comments and editorial track changes to the manuscript. | We thank you for these revisions which the majority have been accepted and incorporated into the revised manuscript. For any specific comments that were included within the manuscript itself, we have included those below along with our responses. | See Introduction |
| Protocol – Introduction | The paragraphs shift abruptly, and the transitions are not smooth. Additionally, the text contains some contradictions. There is a need to provide a more in-depth interpretation of previous findings and the background, followed by a clear explanation of the importance of your study | We revised the text throughout the “Introduction” section to ensure smoother transitions. We have provided a comprehensive overview of previous findings by highlighting the limitations of several key reviews, particularly their focus on specific populations and host countries. | See Introduction |
| Protocol – Introduction | Provide specific examples of cultural challenges or stressors faced by various groups of international students. Cite studies that overlook critical aspects of their experiences. Additionally, provide evidence to support this critique. Many sentences are lengthy and contain multiple ideas, which makes the text harder to follow. Breaking these sentences into focused statements will refine the text. | Based on the reviewer feedback, we incorporated specific examples of cultural challenges and stressors faced by different groups of international students (see section “Introduction”, page 4). Additionally, we have broken down lengthy sentences into more focused statements to enhance clarity and readability throughout “Introduction” section.  With regards to citations from studies that overlook critical aspects of international student experiences, we have referenced relevant reviews in our text (such as systematic reviews by Zhang and Goodson (2011), Li et al. (2014), Brunsting et al. (2018), and Maharaj et al., (2024)) | See Introduction |
| Protocol - Objective and Review Question | Why is this section presented as a separate topic? The objectives and hypotheses are discussed throughout the main text without clear headings or sections. This lack of organisation can lead to confusion for the reader. | The inclusion of Objectives and Review Question as a separate subsection within Introduction ensures that the aims of the study are clearly stated and easily identifiable by readers. To address the reviewer’s concern and ensure smoother transition, we have added the following sentence at the end of the introduction: "To guide this broad exploration of the literature and achieve the goals outlined above, the following objectives and research questions have been established." (see page 8). | To guide this broad exploration of literature and achieve the goals outlined above, the following objectives and research questions have been established. |
| Protocol - Objective and Review Questions | The research questions are too broad and need more focus. Narrowing them will help the address issues in greater depth. Another option is to detail or extend a little bit. The objectives should also be clearer and more precise to give the study a clear path. | We revised the objectives to include the focus on different regional and demographic contexts. We have also revised the research questions to provide clearer focus and examples of mental health concerns, and associated factors (see section “Objective and review questions”, pages 8 and 9). | The objective of this scoping review is to map and summarize research on international students' experiences and concerns (e.g., depression), as well as factors influencing their well-being (e.g., social support and institutional resources.)   1. What mental health and well-being experiences and concerns do international students report? 2. What individual, interpersonal, and environmental factors (e.g., acculturation stress, language barriers, availability of mental health services) are associated with the mental health and well-being of international students, and how do these factors vary across different regions or demographics? |
| Protocol - Eligibility Criteria | The section provides some exclusion criteria, such as excluding studies on short-term exchange students, but it needs to specify other types of studies that may not be relevant, like those focused only on other aspects (e.g. physical health) without focusing on mental health. Since mental health is broadly defined, clarify whether a reference to it in a study is enough for inclusion. Please consider other aspects, like cultural.  The criteria feel scattered throughout the text, making it harder to follow. Streamlining and organising this section more clearly will help readability. Also, some details (e.g. distinction between “college” and “university” terminology) seem unnecessary and don't contribute much to the main objectives. | In accordance with reviewer’s suggestions, we made several changes to the manuscript:   1. We reorganized the inclusion and exclusion criteria to ensure they are clearly defined and logically presented within the relevant sections (see section “Eligibility criteria”, pages 9 to 12). 2. To address the comment regarding mental health definitions, we clarified that studies must mention mental health concerns or experiences to be included in the review (see section “Concept”, pages 10 and 11). The inclusion criteria now specify that eligible studies should focus on aspects such as psychological well-being, emotional well-being, stress levels (including acculturation stress), or mental health status (e.g., depression). Studies focusing solely on physical health without addressing mental health will be excluded. 3. We added references to adjustment and acculturation stress within our inclusion criteria to acknowledge cultural aspects of mental health (see section “Concept”, pages 10 and 11). 4. We removed the distinction between "college" and "university" terminology in the Context section (see pages 11 and 12), to reduce potential confusion. | Population    In terms of inclusion, ...  For the exclusion criteria, ...  Concept  Given the broad definition of mental health, our inclusion criteria will include studies that consider concerns or experiences related to psychological well-being, emotional well-being, stress levels (including acculturation stress), and mental health status (e.g., depression). Studies examining “adjustment” in international students will also be included since this term is closely associated with acculturation stress and reflects how students cope with challenges of studying abroad.  Additionally, if a study primarily focuses on physical health but includes a clear mental health component (e.g. reduction in stress levels), it will also be included in the review.  In terms of exclusion criteria, studies that focus solely on health behaviors that may be related to mental health, such as alcohol use or sleep patterns, without any explicit link to mental health or well-being will not be considered in this review. This exclusion ensures that we do not include studies that may skew our findings by making assumptions about the impact of these behaviors on mental health without direct evidence.  Context  With regards to inclusion criteria, the review will not be restricted by geographical location of the higher education institutions to capture diverse cultural contexts and experiences. This approach will allow us to identify common challenges across different geographical locations, which will enhance relevance and impact of our findings.    For the exclusion criteria... |
| Protocol - Methods | Expand on how these will be integrated or operationalised. This part is important but lacks detail on how all the guidelines and recommendations will work together. | Throughout the protocol, we have referenced the JBI and Levac et al. guidelines to inform our methodology (i.e., use of the PCC framework for developing inclusion and exclusion criteria (see Section “Eligibility criteria”, page 9), use of JBI and Levac et al. in the study selection (see section “Study selection”, page 15) and data extraction processes. For example, we follow the JBI Manual for screening studies and have noted the recommendation from Levac et al. to conduct a pilot extraction on 5-10 articles (see section “Data extraction”, pages 17 and 18). | N/A |
| Protocol - Methods – Sources of Evidence | Why CINAHL? Seems outside the topic. | We have explained the reason for including the CINAHL database in the protocol: CINAHL focuses on nursing and allied health literature, which encompasses research on the mental health of university students pursuing a diverse range of health professional fields. | N/A |
| Protocol - Methods – Sources of Evidence | The selection of databases could be more targeted to the research question by including education-focused and region-specific sources. | We appreciate the reviewer’s suggestion to consider education-focused and region-specific sources for our database selection. We believe that the four databases chosen (MEDLINE, Embase, PsycInfo, and CINAHL) provide a comprehensive range of health research literature that is relevant to our research question. These databases collectively cover various aspects of mental health, institutional factors, and related fields essential for understanding the mental health challenges faced by international students. That said, we acknowledge that incorporating additional databases, including region-specific sources and those pertinent to psychoeducational literature, such as ERIC and Web of Science, could provide a more rounded exploration of the topic. We included this as a limitation in our protocol (see section “Limitations”, pages 20 and 21). | Second, although four databases – MEDLINE, Embase, PsycInfo, and CINAHL – were selected for a range of health research literature on the topic, additional databases, such as region-based, or databases pertinent to psychoeducational literature, including ERIC and Web of Science, could have been considered. As a result, there is a possibility that relevant studies within the psychoeducational domain can be overlooked. |
| Protocol - Methods – Search Strategy | Details on how terms will be adjusted for different databases are important. Also detail sensitivity and specificity. What strategies, boolean, etc? The search strategy appears static, with no indication that the authors will adjust the search strategy based on evolving literature or emerging themes during the screening. Consider using a more flexible approach or strategy. | We added a detailed description of how the search strategy will be tailored to each database at the end of the Sources of Evidence section (see section “Search strategy”, pages 13 and 14). While PsycInfo and Embase will use similar search equations with adjusted descriptors, CINAHL will require amendments for syntax differences (e.g., 'adj' vs. 'N'). An example is now provided to illustrate these adjustments. | This search strategy will be adapted for the three other electronic databases (i.e., Embase, PsycInfo, and CINAHL). While the MEDLINE search equation will be maintained for Embase and PsycInfo, database-specific descriptors will be used to ensure a comprehensive search. For example, MEDLINE may use "exp Students," descriptor while Embase requires a broader set of descriptors, such as "student/ or college student/ or graduate student/ or health student/ or PhD student/ or postgraduate student/ or research student/ or social work student/ or student athlete/ or undergraduate student/ or university student/ or veterinary student/." Similarly, CINAHL will require different descriptors tailored to its indexing system.    Additionally, the MEDLINE search equation will also be adjusted for CINAHL, accounting for differences such as "adj" versus "N" and other variations in search syntax. |
| Protocol - Methods – Study Selection | The use of a small sample for piloting the eligibility criteria may not be representative of the variability in the studies retrieved. Please provide more detailed guidelines on how disagreements will be managed (e.g. for involving the third assessment, documentation, etc.) Expanding the pilot test to include a larger sample (e.g. 20–30 citations) would provide more robust insights into potential challenges in applying the eligibility criteria. | We will use a randomization generator when selecting citations to pilot to ensure a diverse representation of studies. Additionally, we are doing subsets of 10 titles and abstracts at a time, until we reach a 90% agreement rate, following which we will continue to pilot in the same manner for an additional 3 sets to ensure consistency of a minimum of 90% agreement. Thus, while the pilot set is small (n=10) we will be conducting multiple rounds of piloting, allowing for iterative refinement of the criteria. That said, we have included as a limitation in our protocol the point regarding one reviewer completing the titles and abstracts screening following completion of the piloting phase (see section “Limitations”, pages 20 and 21).  Regarding disagreements, our protocol specifies that if uncertainty arises, the first author will discuss with the second reviewer. If consensus cannot be reached, a third reviewer will conduct a full-text review to make the final decision. | Limitations:  Fifth, there is a risk of bias in the selection process given that only one reviewer will be involved in the majority of that process (due to availability of resources). Several strategies are in place to reduce this risk including piloting multiple subsets of 10 articles until a 90% agreement is reached, and continuing to pilot until three additional consecutive sets also results in a minimum of 90% agreement. Moreover, a randomization generator for selecting citations will be employed, increasing the likelihood of obtaining a diverse representation of studies in the pilot. |
| Protocol - Methods – Data Extraction | The proposed charting tool may miss themes important for understanding the main outcomes. Scoping reviews aim to cover a broad literature, including unexpected findings. However, the authors didn’t explain how handle studies outside predefined categories. | We understand reviewer’s concern regarding documentation of unexpected findings. The protocol specifies that the charting tool will remain adaptable to accommodate emergent findings. That said, we have added specification regarding an open-ended category which will ensure that new themes and unexpected data points can be captured (see section “Data extraction”, page 16). | Initially, the legend categories will be informed by the data categories listed in the JBI Manual of Evidence Synthesis, such as: title, Digital Object Identifier (DOI), author(s), year of publication, country of origin, sample size, study design, objective(s), analysis type, and key findings. Furthermore, the categories within the charting tool will be informed by the research questions and the eligibility criteria of the scoping review. The data extraction tool will be finalized upon a detailed review of 10% of the studies to identify additional relevant categories of information to extract. Moreover, to accommodate unexpected themes of information that may arise, open-ended categories will be added, optimizing comprehensive coverage of all relevant data. |
| Protocol - Methods – Data Extraction | The strategy for categorising contextual factors is also too simplified. Higher education varies greatly between countries, and broad categories like "university vs. college" or general won’t gather these complexities. This could limit to reflect the diverse realities of these institutions. Separating data on mental health concerns and assessment methods raises concerns about comparability. Quantitative data can be standardised, but qualitative data requires careful interpretation. The method for integrating both types of data is unclear, which may result in inconsistent analysis of qualitative findings. | We appreciate the reviewer’s feedback and in response, we have clarified our data extraction process as following:   1. For the context component, we will extract information on geographical location and institutional characteristics. Since we will also be extracting data on environmental factors that affect student mental health, such as the adequacy of institutional resources, we aim to avoid redundancy by not duplicating data on the geographical and institutional contexts. To strike a balance, we will focus on extracting key contextual details, including the country and city of the host university, the name of the institution, and relevant institutional characteristics. We added this justification to the manuscript (see section “Data Extraction”, page 17). 2. To address the concern about integrating quantitative and qualitative data, we clarified what data will be extracted and how it could be integrated. We will extract quantitative data, including prevalence rates and scores on mental health scales, to provide standardized metrics. For qualitative data, key themes and observations related to mental health concerns and contributing factors will be extracted, and the frequency of these reports will be counted during analysis. This will allow for some level of integration by presenting both numerical trends from quantitative studies and the prevalence of themes from qualitative studies, ensuring a comprehensive and cohesive understanding of international students’ mental health. | This includes identifying mental health concerns (e.g., depression) and positive experiences (e.g., life satisfaction), along with negative factors affecting mental health (e.g., social isolation) and positive factors contributing to mental well-being (e.g., institutional support).  Quantitative data will include numerical metrics including prevalence rates and scores on mental health scales. For example, if a study reports that 40% of students experienced symptoms of anxiety, this statistic will be extracted.    Qualitative data will focus on key themes or observations related to mental health status and contributing factors. For example, if a study highlights that international students described "stress due to visa challenges" as a common concern or reported "feeling supported by a peer mentorship program," these themes will be extracted as they reflect relevant factors.    In the case of longitudinal studies, data will be extracted at each time point reported in the study (e.g., at the start, mid-way, and completion) to ensure that changes over time are captured. For quantitative studies, numerical data (e.g., prevalence rates) will be collected at each time point. For qualitative studies, evolving factors affecting mental health will be identified, and observations or themes reported by the authors will be extracted separately for each respective time point.  Finally, for the context component of PCC, information pertaining to higher education setting, such as geographical location (e.g., country and community size where the educational institution is located; rural or urban setting), and institutional characteristics (e.g., name of the institution, size of the institution) will be extracted. |
| Protocol – Methods – Data Extraction | The tool collects basic details like sample size and study design, but won’t address how the quality of the studies will be considered. It’s common to not consider this in scoping reviews, but mentioning differences in study designs is important. | We agree it is important to mention differences in study design. Within our manuscript, we have outlined the types of study designs to be included in the extraction tool, based on recommendations by the JBI Manual for Evidence Synthesis. (see section “Data Extraction”, page 16).  The extraction of data on study design will provide readers with context about the methodologies used in the included studies.  Regarding lack of quality assessment, we have addressed this in our limitations section (see page 22). | N/A |
| Protocol – Methods – Data Synthesis and Presentation | The plan for data synthesis and presentation is solid but could be refined with a detailed approach to integrating qualitative data, exploring correlations, and highlighting complex patterns. | We appreciate reviewer’s feedback regarding the data synthesis and presentation plan. In response, we added more detail on how qualitative data will be integrated for each section of the summary, based on the updated methodological guide by Peters et al. (2020) and recommendations by Pollock et al. (2023). Furthermore, we specified that no complex pattern analyses or synthesis will be conducted, which is aligned with scoping review methodology and purpose However, any patterns or associations identified during the extraction process will be summarized and reported descriptively. | The extracted data will be summarized and presented using descriptive statistics (e.g., frequencies and percentages). Tables will be used to visually represent an overview of international student mental health.  The first table will summarize data on types of mental health concerns reported by international students (e.g., depression). Quantitative data (e.g., prevalence rates) and qualitative data (e.g., themes such as "stress due to visa challenges" or "feeling supported by peer mentorship programs") will be presented. For qualitative data, the frequency with which each mental health concern (e.g., depression, anxiety) is mentioned across studies will be counted. For example, if five studies report depression as a concern, it will be noted as a recurring issue in the data. Content analysis will be used to organize multiple types of concerns into categories. Relevant quotes from studies will be included to illustrate the lived experiences of students.  A second table will be used to summarize sociodemographic information extracted from the studies and whether there is an association between certain sociodemographic factors (e.g., age, gender, country of origin, level of education, and field of study) and mental health concerns among international students.  Finally, a third table will include negative and positive factors reported by students that affect their mental health and well-being (e.g., lack of social support). For qualitative data on factors affecting mental health, the frequency with which specific factors are mentioned (e.g., lack of social support, academic pressure) across studies will be organized into categories and counted. Illustrative quotes from included studies will be provided to give context and depth to the reported factors, capturing students' experiences and perspectives. |
| Protocol - Limitations | The limitations section would be stronger by addressing challenges like study quality, publication bias, and heterogeneity in the included studies. | We appreciate the reviewer’s feedback on strengthening the limitations section. We have revised it to address study quality and publication bias limitation (see section “Limitations”, pages 20 and 21). | Additionally, since the review will only include published studies in English and French, there is a risk of publication bias towards western countries. Therefore, certain findings may be overrepresented, thus skewing the overall conclusions from this scoping review.  [...]  Information on study design will be extracted and presented; however, a lack of study quality assessment may reduce reliability of the findings, while at the same time acknowledging that the main purpose of scoping reviews is to map the literature rather than to assess its quality. |
