# Supplementary material for: Mental health concerns and needs of international students in higher education settings: A scoping review protocol
Source: PLoS One. 2025 Aug 8;20(8):e0327812. doi: 10.1371/journal.pone.0327812 (PMC12334020; doi:10.1371/journal.pone.0327812)
Supplement: Attachment — Submitted filename: Response to Reviewers.docx [file pone.0327812.s006.docx]

Dear PLOS ONE Editorial Team

Thank you for your valuable feedback on our protocol. We appreciate the time and effort you have invested in reviewing our submission. Below, we address the comments received from Reviewer #2 and Reviewer #3.

Please note that references to changes made to the manuscript throughout this table correspond to the **Revised Manuscript with Track Changes**, which has been included as part of our resubmission.

| **Reviewer Comment** | **Response** | **Revisions Made** |
| --- | --- | --- |
| Reviewer #2: The Abstract, Under the Methods, “A search strategy guided by the Joanna Briggs Institute Manual (JBI) Manual of Evidence Synthesis”. The first “Manual” should be deleted. | We thank the reviewer for catching this redundancy. The extra word “Manual” has been deleted from the sentence in the “Methods” section of the “Abstract” (see page 2). | A search strategy guided by the Joanna Briggs Institute (JBI) Manual of Evidence Synthesis [18] [...] |
| Reviewer #2: P. 7, at the bottom, “”the Joanna Briggs Institute (JBI) manual of Evidence Synthesis [32]”. “manual” ought to be capitalized. | We thank the reviewer for noticing this. We have corrected the capitalization of “Manual” in reference to the Joanna Briggs Institute (JBI) Manual for Evidence Synthesis on page 8, “Objective and review questions” section | [...] framework proposed by the Joanna Briggs Institute (JBI) Manual of Evidence Synthesis [18] [...] |
| Reviewer #2: P. 7, last full paragraph, “The research questions below have been informed by the objective of the review, as well as the population, concept, and context (PCC) framework proposed by the Joanna Briggs Institute (JBI) manual of Evidence Synthesis [32]” vs. p. 8, under the Eligibility criteria, “The population, concept, and context (PCC) framework proposed by the JBI Manual for Evidence Synthesis [32]”. I feel the two writings are duplicated to a considerable extent. Furthermore, “the population, concept, and context (PCC)” had been expressed too many times throughout the manuscript. | We thank the reviewer for their helpful comment regarding the repeated use of the PCC framework. In response, we revised the manuscript to remove the repetitive use of the population, concept, and context (PCC) framework, while ensuring its appropriate mention where most relevant. | The research questions below have been informed by the objective of the review, as well as guidance by the Joanna Briggs Institute (JBI) Manual of Evidence Synthesis [18] [...]  The population, concept, and context (PCC) framework proposed by the JBI Manual of Evidence Synthesis [18] informed the inclusion and exclusion criteria of this scoping review. |
| Reviewer #3: Multiple citations are listed without clearly linking them to specific claims, and some points are over-cited. Please align each argument with one or two appropriate references and avoid excessive citation. | We revised the manuscript, particularly “Introduction” section (see pages 3 to 8), to reduce citation density by selecting the most relevant examples. | See Introduction |
| Reviewer #3: Please explicitly describe the inclusion and exclusion criteria (e.g., age range, undergraduate/graduate status, study period, countries targeted). | We acknowledge the reviewer’s concern regarding the clarity of the inclusion and exclusion criteria for the student population.  As originally stated, this review does not impose restrictions based on sociodemographic characteristics such as age, gender, level of study, or nationality, in order to support a broad and inclusive understanding of international student mental health.  To address the reviewer’s suggestion more directly, we have revised the “Population” subsection of “Eligibility criteria” (see page 10) to explicitly list these criteria and clarify our approach to inclusion and exclusion. Additionally, we have added a table to the Eligibility criteria section (“Table 1. Inclusion and exclusion criteria”; see pages 9 and 10) that summarizes the inclusion and exclusion criteria across all four domains (Population, Concept, Context, and Types of Sources of Evidence) to further enhance transparency and clarity. | [..] studies that focus on international student populations enrolled in short-term exchange studies abroad (typically lasting less than one year) will be excluded [...]  The review will not be restricted by nationality, citizenship, age, gender, sexuality, or level of study, to allow for a broad understanding [...]  See “Table 1. Inclusion and exclusion criteria” for details (pages 9 and 10). |
| Reviewer #3: There is an inconsistency: while the section mentions two reviewers, it later states that the first author alone will screen records. Please clarify exactly who performs each step (independently or jointly), and explain the role of automation tools like EndNote and Covidence. It is also better to involve at least two reviewers or include a cross-checking mechanism to ensure accuracy and reduce bias. | We thank the reviewer for this helpful observation. We revised the manuscript to clarify the roles of each reviewer throughout the screening process, as well as the use of software tools (EndNote, Covidence, and Excel) at different stages (see “Study selection” section, pages 16 and 17).  As recommended by the JBI Manual for Evidence Synthesis, the screening will begin with a pilot test in which two reviewers (the first author and a member of the research team) independently screen subsets of 10 citations to ensure consistency in applying the eligibility criteria. Once a 90% agreement rate is achieved, the full screening process will be carried out by one reviewer—the first author. In cases of uncertainty, the first author will consult with a second reviewer, and if needed, a third reviewer (senior author) will be involved in reaching consensus.  We acknowledge that dual independent screening throughout would strengthen the process; however, due to resource limitations, only one reviewer will conduct the majority of screening. This limitation has already been noted in the manuscript (see “Limitations” section, page 23). | See Study selection |
| Reviewer #3: Please define any rules for handling different time points. Please state how the team will deal with incomplete or ambiguous data (e.g., contact authors, make assumptions). | We thank the reviewer for these important suggestions.  Regarding study findings with multiple time points, our manuscript already specifies that data will be extracted at each time reported (e.g., start, mid-point, and completion) to capture changes over time (see “Data extraction” section, pages 18 and 19). No further changes were made in response to this point.  In response to reviewer’s concerns about the incomplete or unclear information, we added a statement clarifying that assumptions will be made where appropriate and in consultation with a second reviewer (senior author). If clarification is not possible, only the data explicitly presented in the publication will be extracted (see “Data extraction” section, page 19). | For incomplete or unclear information, assumptions will be made where appropriate and in consultation with the senior author. These assumptions will be specified in the report, so that it is clear to the reader where any assumptions were made. If clarification is not possible, only the data explicitly reported in the publication will be extracted. |
| Reviewer #3: Please note if a risk of bias assessment is omitted and explain why, in accordance with scoping review standards. | We appreciate the reviewer’s comment regarding risk of bias assessment. As per the PRISMA Extension for Scoping Reviews (PRISMA-ScR) Checklist (Tricco et al., 2018), formal risk of bias or quality appraisal is not required in scoping reviews, as their primary aim is to map the breadth and nature of existing literature rather than to evaluate methodological quality. As such, no risk of bias assessment will be conducted.  Although this has already been acknowledged in the “Limitations section” (see pages 23 and 24) of the manuscript, we added a corresponding statement in the “Data extraction” section (see page 19) to ensure clarity and avoid further confusion. | Consistent with scoping review methodology and the PRISMA-ScR guidelines [23], no formal risk of bias or certainty appraisal (e.g., GRADE assessment [27]) of the included studies will be conducted, as the purpose of this review is to map the available literature rather than assess individual study rigor. The extracted study design information will be presented to help the readers interpret the evidence within its methodological context. |
| Reviewer #3: Please state that certainty assessments (like GRADE) are not conducted in this scoping review and provide a rationale. | We added a clarifying statement (see “Data extraction section”, page 19) in response to this comment to explicitly note that no formal certainty appraisal (GRADE) will be conducted, consistent with PRISMA-ScR Checklist (Tricco et al., 2018). | Consistent with scoping review methodology and the PRISMA-ScR guidelines [23], no formal risk of bias or certainty appraisal (e.g., GRADE assessment [27]) of the included studies will be conducted, as the purpose of this review is to map the available literature rather than assess individual study rigor. The extracted study design information will be presented to help the readers interpret the evidence within its methodological context. |
| Reviever #3: The sentences of “The transition… signifies” were unnatural and lengthy. Please use concise expressions like, “Transitioning to higher education brings academic, personal, and emotional challenges.” | We revised the manuscript, particularly “Introduction” section (see pages 3 to 8), to improve clarity and conciseness. The original sentences have been rephrased to use more straightforward language in line with the reviewer’s recommendation. | See Introduction |
| Reviewer #3: What are individual, interpersonal, and environmental factors... are associated with...” should be correct to: “What individual, interpersonal, and environmental factors are associated with…” to fix the grammatical error. | We appreciate the reviewer pointing out this grammatical error. It has been corrected as suggested (see section “Objective and review questions”, page 8) | What are individual, interpersonal, and environmental factors (e.g., acculturation stress, language barriers, availability of mental health services) are associated [...] |
| Reviewer #3: While databases are listed, the date of last search for each source is not provided. Please indicate the search date for each database (e.g., “searched on March 5, 2024”) to ensure transparency. | We thank the reviewer for this observation. We have now added the specific date ranges for all database searches in the “Search Strategy” section (see page 15) of the manuscript to ensure transparency. | The final searches for MEDLINE, Embase, and PsycInfo were conducted on March 27, 2024; the CINAHL search was conducted on April 4, 2024. |
